# Does behavioural parent training reduce internalising symptoms (or not) among children with externalising problems? Systematic review and meta-analysis

**DOI:** 10.1007/s00787-022-02122-3

**Published:** 2022-12-17

**Authors:** Christy Bloss, Sophie Brown, Vilas Sawrikar

**Affiliations:** 1 Department of Clinical Psychology, School of Health in Social Science, University of Edinburgh, Edinburgh, UK; 2 Centre of Applied Developmental Psychology, University of Edinburgh, Edinburgh, UK

**Keywords:** Behaviour parent training, Internalising, Externalising, Comorbidity, Transdiagnosis

## Abstract

Behaviour parent training (BPT) is known to effectively reduce child externalising problems. However, evidence for BPT to have secondary benefits for reducing internalising symptoms remains unclear. To address this, electronic databases (PsycINFO, MEDLINE, EMBASE, CINAHL, and SCOPUS) were systematically searched for studies examining internalising outcomes from BPT among children aged 2–12 years with clinically elevated externalizing problems. Outcomes for internalising problems following BPT were analysed by meta-analysis. Of 9105 studies identified, 24 studies met the eligibility criteria. Results from meta-analysis demonstrated a significant small treatment effect size (*g* = − 0.41) for reducing internalising symptoms immediately after treatment. Studies showed moderate heterogeneity (*I*^2^ = 44%). Moderation analyses indicated that the overall treatment effect was robust against variations in treatment and study design characteristics. However, a review of individual study methods indicate that these results are limited by significant heterogeneity and limitations in clinical assessment. Overall, the results suggest that BPT programmes for reducing externalising problems have the potential to improve internalising outcomes, but that there is limited information to determine the reliability of these effects, highlighting the need for further investigation.

**Supplementary Information:**

The online version contains supplementary material available at 10.1007/s00787-022-02122-3.

## Introduction

Children with externalising problems have difficulties with noncompliance, tantrums, aggression, and anger, which become problematic when they are in greater frequency than expected for the child’s developmental stage [1]. Notably, early onset externalising problems emerging in childhood are linked to poor long-term psychosocial outcomes, alluding to the significant burden of disease associated with externalising problems [2–4]. As such, disseminating evidence-based treatments for children with externalising behaviours has become a high priority focus for applied mental health researchers and clinicians [4, 5]. In line with this, behavioural parent training (BPT) is recognised as an evidence-supported treatment (EST) for externalising problems in childhood [6, 7], and there is growing interest in upscaling BPT as a strategy for early intervention, prevention, and treatment of child problems across the population [8].

An outstanding research question relates to how BPT should be delivered to meet the mental health needs of children with multiple problems. For instance, comorbid internalising problems are particularly common among externalising children, afflicting 20–35% of children in the community and up to 75% of children referred to clinical services [9]. However, there is little clinical guidance available for children with co-occurring internalising and externalising problems [10]. Internalising symptoms are associated with avoidant and withdrawal behaviour related to fear- and distress-related stimuli, which contrasts with approach-related behaviour underpinned by anger and aggression in externalising problems [11, 12]. As such, ESTs for internalising problems are generally evaluated separately creating boundaries between conditions which don’t reflect real-world clinical populations where comorbidity is the rule rather than exception [13]. In research examining practices adopted by practitioners, therapists report emphasising the treatment of externalising over internalising problems, but the justification for doing so is unclear [14]. Importantly, comorbid internalising and externalising problems may be a key indicator of prognosis. Research suggests these children display greater clinical severity and impairment that potentially leads to poor response to ESTs [15, 16]. On the contrary, research has shown that treatment of externalising problems may be unaffected by comorbid internalising symptoms [17, 18]. The mixed findings emphasise the need to evaluate BPT in relation to internalising outcomes to inform optimal treatment delivery in context of common comorbidities. Previous research has attempted to address this by initially evaluating whether BPT may have crossover effects in improving internalising symptoms, but results are inconsistent [19, 20]. The aim of the current meta-analysis was to systematically review outcomes of controlled studies to understand how reliable BPT is in reducing internalising symptoms following treatment.

BPT programmes draw from social-ecological theory and research emphasising negative family environments in the development of externalizing problems [21–23]. The skills taught in BPT are based largely on Patterson’s ‘coercion’ model, which applies social learning theory in explaining the development of externalising behaviours. The model hypothesises coercive parent–child interactions occurring in the context of child externalising behaviours act as negative reinforcement traps, whereby escalatory behaviours by the child are negatively reinforced when it extinguishes parent’s attempt to manage the child, while parent capitulations are negatively reinforced by the extinction of child’s escalatory behaviour. Parents are also known to show less positive affect and involvement that reinforces child positive behaviour. Parents are therefore taught procedures to increase reinforcement for positive behaviours and discipline-focused strategies to set limits on negative behaviour to break the coercive cycles reinforcing externalising behaviour [24]. The most evaluated BPT programs include Incredible Years [25], Parent–Child Interaction Therapy [26] and Triple P-Parenting Program [27]. These programs can be delivered in a group-based or individual format, and include activities with children, video feedback or live demonstrations from therapists. A large body of evidence supports BPT as a standalone treatment for children with externalising disorders in early-to middle-childhood (2–12 years) which meets the criteria for it being considered an EST for these problems [28–30].

One line of research has inquired whether BPT as a standalone treatment may have clinical utility in reducing internalising problems, suggesting it represents an intervention format which benefits children with externalising and internalising problems [10, 11, 31]. These suggestions are based on theory emphasising the salience of transdiagnostic and contextual processes underpinning the progression of externalising and internalising problems [32–35]. The Dual Failure Model [33], for instance, proposes that children with externalising problems are more likely to experience internalizing problems, both concurrently and prospectively, due to concomitant negative family environments and social and academic difficulties. To that end, parental rejection, withdrawal, and disengagement, which are closely related to lower positive affective parenting behaviours, represent etiological and maintaining factors of internalising problems for children with externalising behaviours. Findings from empirical studies show that poor relationships with parents among externalising children represents a vulnerability risk factor for depressed mood in adolescence [36, 37]. Elsewhere, research examining parent–child interactions in families of children with anxiety have identified maladaptive, coercive parent–child interactions also reinforce child anxiety like those for externalising behaviour [38, 39]. This research putatively suggests targeting negative parenting behaviour in BPT may have cross over benefits for internalising symptoms. However, cross over benefits may be limited since well-established treatment components for internalising problems such as cognitive restructuring and exposure are not included in BPT. Considering this uncertainty, it is timely to interrogate the evidence by meta-analysis and benchmark average treatment effects against recommended treatments for child internalising problem to evaluate whether BPT could be putatively viewed as a quasi-universal psychological intervention format for children with externalising and internalising symptoms (c.f., cognitive-behavioural treatment for child internalising problems show moderate-large treatment versus control effect sizes at post-treatment: anxiety, *g* = 0.76, depression, *g* = 0.79; [40]).

Two recent systematic reviews provide mixed support for BPT reducing internalising symptoms among children with externalising problems following treatment. Buchanan-Pascall et al. (2018) [20] conducted a systematic review and meta-analysis of randomised control trials (RCTs) evaluating the effects of group-based BPT on internalising symptoms among children aged 4–12 years. One in four studies reported statistically significant improvements in group BPT over control groups, and pooled effects indicated only marginally non-significant small effect size (*d* = − 0.18; *p* value = 0.056). Recently, Zarakoviti et al. [19] conducted a systematic narrative review of RCTs with specific reference to the IY and Triple P programs for children with externalising disorders aged 2–10 years. Results indicated that two-thirds of studies found statistically significant improvements in internalising symptoms post-treatment. Taken together, these reviews provide some preliminary evidence for BPT to reduce internalising symptoms following treatment. However, significant treatment effects for reducing internalising symptoms were not consistently identified within or across reviews. These reviews were also largely based on group interventions and therefore excluded assessment of individual based BPT. These reviews also did not consider baseline levels of internalising symptoms, which would help differentiate outcomes for children with comorbid presentations versus externalising problems only. Finally, previous reviews did not conduct moderation analysis to determine the putative influence of treatment and study characteristics on outcomes. These limitations suggest the effects of BPT on internalising problems for children with externalising problems in childhood warrants further examination by meta-analytic review.

The current meta-analysis was aimed at evaluating the pooled treatment effect on internalising outcomes in relation to both individual and group-based BPT for children with externalising problems. First, it was hypothesised that BPT will be associated with significant pre- to post-treatment reductions in internalising symptoms overall. Subsequently, the current review examined whether treatment effects were moderated by: (1) child age, early childhood anxiety versus later childhood; (2) baselines levels of internalising problems, children with elevated internalising symptoms versus low internalising symptoms; (3) measurement methods, since different instruments adopt various scales to calculate symptom severity, (4) treatment format, individual versus group-based BPT, (5) programme type (e.g., TP vs IY), and (6) risk of bias (any risk vs no risk of bias). It was hypothesised that secondary benefits of BPT for internalising symptoms were reliable and, therefore, the size and significance of the treatment effect would not be moderated by these characteristic differences across studies. The influence of measurement methods and programme type was examined post-hoc since there was no a-priori knowledge of measures or programmes used in research studies.

## Methods

### Search strategy

An electronic search was completed in February 2022 which aimed to identify peer-reviewed studies evaluating BPT targeting externalising behavioural problems for children aged between 2 and 12 years. The databases PsychINFO, Embase, Medline, CINAHL and Scopus were systematically searched using the following search terms: (child* OR toddler OR adolesc*) AND (conduct disorder OR oppositional defian* disorder OR behavio* problem* OR externali*) AND (parent* program* OR parent* intervention OR parent* group OR parent training OR parent management training) AND (depressi* OR anxiety OR mood dysregulation OR internali* OR co-morbid* OR co-occur* OR co-exist*).

### Study selection

Studies met criteria to be included if they: (a) included children aged between 2 and 12 years old; (b) the children were assessed as having diagnosed or subthreshold externalising problems and/or a mean score in the clinical range on a standardised validated measure of externalising behaviours, and/or who were reported by parents or referred to services for elevated externalising symptoms; (c) evaluated a therapist led parenting programme that had core components based on social learning theory [22]; (d) evaluated BPT against a waitlist, treatment as usual, or active control group within an RCT design; and (e) included a pre- and post-treatment measure of child internalising problems.

Children with intellectual disabilities, global developmental delay, communication disorders or autism spectrum disorders were excluded. BPT programmes with adjunct components (e.g., child, teacher, or self-directed component) were excluded as the aim of the review was to examine the effects of parent directed BPT only. Moreover, studies were excluded if the parenting programme was digital/online or self-directed, the study was not an RCT, or published in non-English journals.

Study selection was independently performed by three reviewers in Covidence [41]. First, title and abstracts were screened by two of the reviewers using an abstract screening tool based on the inclusion and exclusion criteria (see Table 1 in supplementary information). The third reviewer resolved disagreement in this abstract screening phase. In the full text review stage, all study eligibility criteria were applied, and all three reviewers independently assessed each study for inclusion. Studies required consensus from two reviewers to be included, and any conflicts were discussed and then resolved by the third reviewer.

### Data extraction

Data extraction was independently completed by two reviewers using Higgins and Green’s [42] proforma developed for systematic reviews and meta-analysis. Study characteristics comprising of participants, interventions, comparators, and internalising outcomes were synthesised. The third reviewer resolved any discrepancies and reviewed the accuracy of extraction. Data extraction for the meta-analysis was performed by one reviewer, while the other reviewers checked the extracted information for completeness and accuracy.

### Assessment of study quality: risk of bias

All studies in this review were assessed using the Cochrane Collaboration’s risk of bias assessment tool [43]. Each RCT was assessed in seven domains: random sequence generation, allocation concealment, blinding of participants and personnel, blinding of outcome assessors, incomplete outcome data, selective reporting, and other sources of bias. Assessments of high (bias likely to influence the confidence in results), low (bias unlikely to influence the confidence in results) or unclear (doubt of influence in the results) bias were made by two of the reviewing authors. Final ratings for each domain were determined by consensus between raters.

### Synthesis of results and meta-analysis

Narrative synthesis of study characteristics and risk of bias was first reported to contextualise findings from included studies. A subsequent synthesis of results across studies evaluated whether BPT was statistically significantly associated with reductions in internalising symptoms following treatment. Results were based on reported significance of pre-to post-treatment differences between BPT versus control group. Follow-up outcomes were reported if details of immediate post-treatment outcomes were not reported. Table 1 summarises study characteristics. An overview of study findings is presented in Table 2.

**Table 1:** Summary of study characteristics

Study	Parenting Programme(s)	Country	Conditions	Participants	Delivery format	Programme Description
Abrahamese et al. 2016 [50]	Parent–Child Interaction Therapy (PCIT)	Netherlands	PCIT (*n* = 20) FCT (*n* = 25)	Parents of children (2–8 years) referred over concerns about disruptive behaviour	Individual	Parental didactic session followed by weekly coaching sessions of 1 h
Altafim et al. 2019 [51]	ACT Raising Kids Safe Parenting Programme	Brazil	PT (*n* = 61) WLC (*n* = 61)	Parents of children (3–8 years) with elevated behavioural problems	Group	Eight 2 h group sessions once per week, max 10 mothers per group
Axberg and Broberg 2012 [52]	Incredible Years Parenting Program (IY)	Sweden	IY (*n* = 38) WLC (*n* = 24)	Parents of children (4–8 years) with diagnosed ODD	Group	2 h group sessions for 12–14 weeks
Bagner et al. 2010 [59]	Parent–Child Interaction Therapy (PCIT)	USA	PCIT (*n* = 14) WLC (*n* = 14)	Mothers of children aged 1.5–5 years, born < 37 weeks with a clinically significant CBCL externalising score	Individual	Once a week for 1 h
Braet et al. 2009 [53]	Parent Management Training (PMT)	Belgium	PMT (*n* = 34) WLC (*n* = 30)	Parents of children (4–7) years with early onset behavioural problems	Group	11 sessions over 24 weeks. 2 h sessions with groups of 8 to 10 parents
Herman et al. 2011 [64]	Incredible Years Parenting Program (IY)	USA	IY (*n* = 31) WLC (*n* = 26)	Mothers of children (4–8 years) who met DSM-IV criteria for ODD	Group	22–24 weekly, 2 h group sessions. Each group had 10 to 12 parents
Keown et al. 2018 [65]	Low intensity culturally adapted Triple P-Positive Parenting Program (Te Whānau Pou Toru)	New Zealand	TP (*n* = 41) WLC (*n* = 29)	Parents of children (3–7 years) referred over behavioural concerns	Group	Two × 2 h parenting discussion groups with 4 to 12 parents per group
Kjobli and Ogden 2012 [55]	Brief Parent Training (BPT)	Norway	BPT (*n* = 108) CG (*n* = 108)	Parents of children (3–12 years) at an early stage of problem behaviour development or had developed conduct problems	Individual	Short-term intervention (3–5 sessions) lasts approximately 3–5 h
Kjobli et al. 2013 [66]	Parent Management Training, the Oregon model (PMTO)	Norway	PT (*n* = 72) WLC (*n* = 65)	Parents of children (3–12 years) who exhibited conduct problems	Group	12 weekly sessions lasting 2.5 h with max 16 participants
Larsson et al. 2009 [61]	Basic Incredible Years Parenting Program (IY)	Norway	IY(*n* = 51) WLC (*n* = 30)	Parents of children (4–8 years) with sub-threshold or diagnosed ODD and or/CD	Group	2 h sessions, 12–14 weeks, 10–12 parents per group
Lau et al. 2011 [67]	Incredible Years Parenting Program (IY)	USA	IY (*n* = 32) WLC (*n* = 22)	Parents of children (5–12 years) referred due to behavioural concerns or parental discipline	Group	14 sessions lasting 2 h, 5–10 parents per group
Leckey et al. 2019 [56]	Incredible Years Parenting Program (IY)	Ireland	IY (*n* = 19) WLC (*n* = 14)	Parents of children (3–7 years) referred for persistent hyperactivity, inattention and/or impulsive behaviours	Individual	20 weekly 2–2.5 h sessions
Leung et al. 2017 [57]	Parent- Child Interaction Therapy (PCIT)	China	PCIT (*n* = 32) WLC (*n* = 32)	Parents of children (2–7 years) diagnosed ADHD with a score above the cut-off on the ECBI	Individual	Once per week for 1 h
Little et al. 2012 [68]	Triple P- Positive Parenting Program (TP)	United Kingdom	TP (*n* = 73) WLC + SAU (*n* = 73)	Parents of children (4–9 years) with symptoms of a behavioural or social-emotional disorder, scoring above the “high need” threshold on the SDQ total difficulties score	Group	Four 2.5 h parent sessions plus four individual telephone consultations (15–30 min duration each)
Mersky et al. 2016 [58]	Parent- Child Interaction Therapy (PCIT)	USA	Extended PCIT (*n* = 19) Brief PCIT (*n* = 39) WLC-SAU (*n* = 33)	Foster parents of children (3–6 years) in the clinical range for externalising problems on the ECBI	Group	Two full-day PCIT trainings and 8 weeks of phone consultation. Additional booster training and 6 weeks consultation for extended PCIT group. Trainings consisted of between 4 and 8 parent–child dyads
Morawska and Sanders 2009 [69]	Triple P – Positive Parenting Program (TP)	Ausralia	TP (*n* = 37) WLC (*n* = 38)	Parents of gifted children (3–10 years) who had reported concerns about their child’s behaviour	Group	Five weekly, 2 h group sessions, then three weekly, 15 min telephone consultations and a final 2 h group session
Morpeth et al. 2017 [70]	Incredible Years Parenting Program (IY)	United Kingdom	IY (*n* = 110) WLC (*n* = 51)	Parents of children (3–4 years) who scored above the total difficulties clinical cut-off score on the SDQ	Group	12 × 2 h group sessions once a week with a max of 12 parents per group
Scavenius et al. 2020 [75]	Parent Management Training, the Oregon model (PMTO)	Denmark	PT (*n* = 64) SAU (*n* = 62)	Parents of children (4–12 years) who were referred over behavioural problems	Individual	1-h sessions on a weekly basis On average, treatment consisted of 23 individual sessions over the course of 7 months
Scott, 2005 [76]	Incredible Years Parenting Program (IY)	United Kingdom	IY (*n* = 59) WLC (*n* = 37)	Parents of children (3–8 years) who were referred for antisocial behaviour to child mental health service	Group	13–16 × 2 h group session. Intervention sessions were videotaped, and weekly supervision meetings were held to ensure adherence to the manual
Shimaburkuro et al. 2020 [74]	Well Parent Japan (WPJ)	Japan	WPJ (*n* = 28) WLC (*n* = 24)	Parents of children (6–12 years) displaying 6 or more symptoms of ADHD	Group	13 × 2 h group sessions. WPJ is a Japanese language adaptation of the New Forest Parenting Programme for ADHD. Included sessions to improve mother's psychological wellbeing
Thomas and Zimmer-Gembeck, 2012 [77]	Parent–Child Interaction Therapy (PCIT)	Australia	PCIT (*n* = 61) WLC (*n* = 91)	Parents of children (3–7 years) at high risk or engaged in maltreatment with significant behavioural problems	Individual	12–16 coaching sessions and 2 assessment sessions
Webster-Stratton and Herman 2008 [73]	Incredible Years Parent Training (IYPT)	USA	IY (*n* = 111) WLC (*n* = 70)	Parents of children (3–8 years) who had an ECBI score > 2 SD above normal cut off	Group	14 group sessions
Wiggins et al. 2009 [71]	Pathways Triple P—Positive Parenting Program (TP)	Australia	TP (*n* = 30) WLC (*n* = 30)	Parents of children (4–10 years) with borderline to clinically significant relationship disturbance and child behavioural problems	Group	9 weekly 2 h group sessions
Yusuf et al. 2019 [72]	Triple P- Positive Parenting Program (TP)	Turkey	TP (*n* = 30) WLC (*n* = 30)	Parents of children (7–12 years) with diagnosed ADHD	Group	5 × 2 h group sessions and 3 × 15–30 min individual telephone calls

*CG control group, *PT* parent training, *WLC* waitlist control, *SAU* service as usual, *FCT* family creative therapy, *n* number of children randomised

**Table 2:** Summary of internalising outcomes across studies

Study	Groups	Child internalising outcomes
Abrahamese et al. 2016 [50]	PCIT (*n* = 12) FCT (*n* = 13)	PCIT = FCT CBCL-IS (pre-post, *p* = 0.054^a^, *d* = − 0.83)
Altafim et al. 2019 [51]	ACT (*n* = 40) WLC (*n* = 41)	ACT = WLC SDQ—IS (pre-post, *p* = 0.15, *η*2 = 0.026)
Axberg and Broberg 2012 [52]	IY (*n* = 34) WLC (*n* = 20)	IY = WLC SDQ emotional symptoms (pre-post, *p* = 0.993, *d* = 0.004)
Bagner et al. 2010 [59]	PCIT (*n* = 11) WLC (*n* = 14)	PCIT > WLC CBCL-IS (pre-post, *p* = 0.000**^a^, *d* = 1.4)
Braet et al. 2009 [53]	PMT (*n* = 30) WLC (*n* = 19)	PMT = WLC CBCL-IS (pre-post, *p* > 0.05^a^, *d* = 0.23)
Herman et al. 2011 [64]	IY (*n* = 29) WLC (*n* = 26)	IY = WLC CBCL-IS (pre-post, *p* = 0.06, *d* = 0.42)
Keown et al. 2018 [65]	TP (*N* = 41) WLC (*N* = 29)	TP = WLC SDQ emotional symptoms (pre-post, *p* = 0.117^a^, *d* = 0.45)
Kjobli and Ogden 2012 [55]	BPT (*N* = 108) CG (*N* = 108)	BPT > CG CBCL anxiety/depression (pre-post, *p* = 0.04*^a^, *d* = 0.29)
Kjobli et al. 2013 [66]	PMTO (*N* = 72) WLC (*N* = 65)	PMTO = WLC CBCL anxiety/depression (pre-post, *p* = 0.13 ^a^, *d* = 0.26)
Larsson et al. 2009 [61]	Mothers – IY (*N* = 45) WLC (*N* = 28) Fathers – IY (*N* = 25) WLC (*N* = 21)	IY > WLC Mothers CBCL-IS (pre-post, *p* < 0.05* ^a^, *d* = 0.57) Fathers CBCL-IS (pre-post, *p* > 0.05 ^a^, *d* = − 0.07)
Lau et al. 2011 [67]	IY (*N* = 30) WLC (*N* = 20)	IY > WLC CBCL-IS (pre-post, *p* < 0.05* ^a^, *d* = − 0.51)
Leckey et al. 2019 [56]	IY (*N* = 19) WLC (*N* = 14)	IY = WLC SDQ emotional symptoms (pre-6 months fu, *p* > 0.05, d = − 0.29)
Leung et al. 2017 [57]	PCIT (*N* = 32) WLC (*N* = 32)	PCIT > WLC CBCL-IS (pre-post, *p* < 0.001** ^a^, partial *η*2 = 0.280–0.449)
Little et al. 2012 [68]	TP (*N* = 73) WLC/SAU (*N* = 73)	TP = WLC SDQ emotional symptoms (pre-6 months fu, *p* = 0.83)
Mersky et al. 2016 [58]	Extended PCIT (*n* = 15) Brief PCIT (*n* = 31) WLC/SAU (*n* = 29)	Extended PCIT + Brief PCIT = SAU/WLC (quadratic pairwise contrast) CBCL-IS (pre-post, *p* = 0.735^a^, *r*^2^ = 0.00) Extended PCIT + Brief PCIT > SAU/WLC (linear pairwise contrast) CBCL-IS (pre-post, *p* = 0.002** ^a^, *r*^2^ = 0.08)
Morawska and Sanders 2009 [69]	TP (*N* = 33) WLC (*N* = 37)	TP = WLC SDQ emotional symptoms (pre-post, *p* = 0.925^a^)
Morpeth et al. 2017 [70]	IY (*n* = 110) WLC (*N* = 51)	IY = WLC SDQ emotional symptoms (pre- 6 months fu, *p* > 0.05)
Scavenius et al. 2020 [75]	PMTO (*n* = 65) SAU (*n *= 59)	PMTO = SAU SDQ-IS (pre-post, *p* > 0.05^a^)
Scott 2005 [76]	IY (*n* = 58) WLC (*n* = 26)	IY > WLC SDQ emotional symptoms (pre-12 month fu, p = 0.029*, *d* = 0.30)
Shimaburkuro et al. 2020 [74]	WPJ (*n* = 27) WLC (*n* = 21)	WPJ > WLC CBCL-IS (pre-post, *p* = 0.012* ^a^, *d* = 0.774)
Thomas and Zimmer-Gembeck 2012 [77]	PCIT (*N* = 61) WLC (*N* = 91)	PCIT > WLC CBCL-IS (pre-post, *p* = 0.014*, *d* = − 0.30)
Webster-Stratton and Herman 2008 [73]	Mothers—IY (*N* = 111) WLC (*N* = 70) Fathers—IY (*N* = 103) WLC (*N* = 67)	Mother – IY > WLC, Father – IY = WLC Mother – CBCL-IS (pre-post, *p* < 0.05*, *d* = 0.37) Father – CBCL-IS (pre-post, *p* = 0.16, *d* = 0.26)
Wiggins et al. 2009 [71]	TP (*N* = 30) WLC (*N* = 30)	TP > WLC CBCL-IS (pre-post, *p* = 0.025*, partial *η*2 = 0.084)
Yusuf et al. 2019 [72]	TP (*N* = 23) WLC (*N* = 25)	TP > WLC SDQ emotional symptoms (pre-post, *p* = 0.004*, *d* = 0.99)

*SDQ-IS strengths and difficulties questionnaire-internalising subscale, *CBCL-IS* child behaviour checklist-internalising subscale, *partial η2 partial eta squared* η2 eta squared, *d* Cohen’s d, *r*^*2*^ R squared, *WLC* waitlist control, *SAU* service as usual

> Indicates a statistically significant difference in the internalising score between intervention versus control, where *p < 0.05 and **p < 0.001

= Indicates a non-statistically significant difference in the internalising score between intervention versus control

^a^Finding relates to within-group changes in symptoms from pre-post. Number of months follow-up (months fu) was reported for relevant studies

Meta-analysis was subsequently conducted in Review Manager version 5.4 [44]. Data from measures of child internalising symptoms were combined in a meta-analysis using a random effects model because of expectations of heterogeneity in effect sizes related to different study characteristics [45, 46]. Standardised mean differences (SMDs) were calculated and combined using the inverse variance method to give an overall estimate effect using Hedges’ g. The significance of Hedges’ g was evaluated at *α* = 0.05. The SMD referred to the standardised difference in internalising scores between BPT and control group at post-treatment assessment (i.e., average score of the control group minus average score for the BPT treatment group, divided by pooled standard deviation of the two groups). In studies that reported standard errors (SEs), the standard deviations (SDs) were calculated using the Cochrane formula [42]. An effect of 0.2 indicated a small effect size, 0.5 indicated a moderate effect size, and 0.8 indicated a large effect size [47]. Forest plots and the *I*^2^ index were visually inspected for assessment of heterogeneity. Interpretation of the *I*^2^ index was 0%: homogenous, 25%: small heterogeneity, 50% moderate heterogeneity, and 75% as large heterogeneity. We also reported 95% prediction intervals in assessing heterogeneity in accordance with recommendations by Borenstein et al. [48]. The funnel plot alongside Egger’s test of publication bias [49] was used to assess publication bias.

Subgroup analysis was used to determine whether calculations of effect sizes were moderated by child, treatment, and/or study characteristics. This was done by coding studies into the following subgroups for comparisons: (1) child age, early childhood (mean age of child in study ≥ 2 and ≤ 6) versus later childhood (mean age of child in study ≥ 7 and ≤ 12); (2) baseline internalising symptoms, high comorbid internalising (average internalising baseline score was in the borderline or clinical range) versus low internalising (average internalising baseline score in the normal range), (3) treatment format, individual versus group based BPT, (4) measure used (e.g., CBCL vs SDQ), and (5) programme type (where there were multiple studies to compare (e.g., TP vs IY vs PCIT). Treatment effects were estimated for each subgroup in Revman version 5.4, and moderation was evaluated by the significance of the difference between the estimated effect for each subgroup.

## Results

### Literature search

**Fig. 1:**
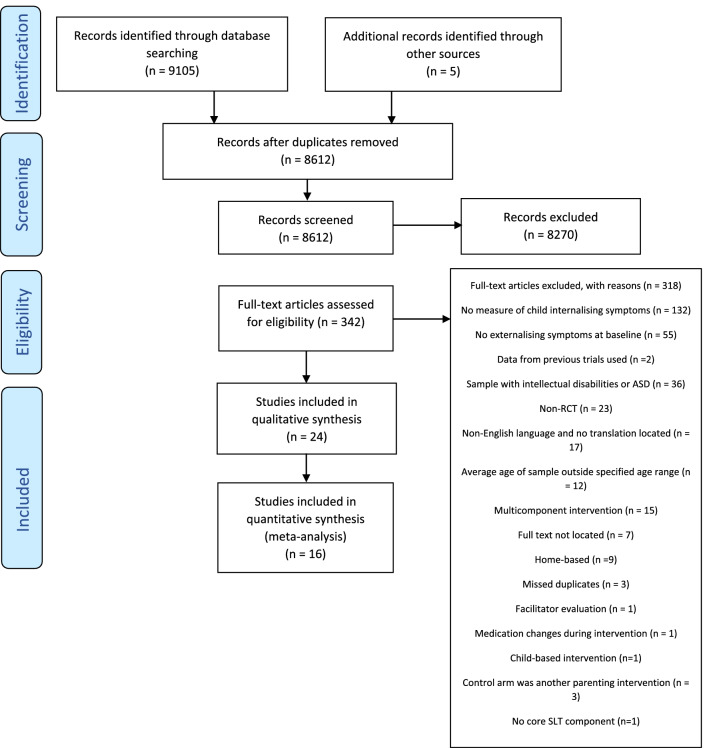
PRISMA flow diagram describing identification and selection of studies for inclusion in the systematic review

Figure 1 outlines the PRISMA flow diagram describing identification and selection of studies for inclusion in the systematic review. The initial database search identified 9105 studies with 5 additional studies identified through searches of previous studies’ bibliography. From these, 493 duplicate studies were removed, while 8270 studies were deemed to have not met the eligibility criteria based on reviewing abstracts and titles, leaving 342 studies eligible for full text review. Inter-rater agreement between the two primary reviewers of inclusion of studies based on abstracts and titles was 96.6% (see supplementary information Tables 2 and 3). A further 318 studies were subsequently excluded at the full text review stage based on applying inclusion/exclusion criteria (see Fig. 1 for reasons). Inter-rater agreement between the two primary reviewers of inclusion of studies based on full texts was 93.8%. In total, 24 full text RCTs were identified as eligible. Publication of these studies were between 2005 and 2020.

### Study characteristics

The characteristics for each study are summarised in Table 1. The pooled sample size of participants across 24 studies was *N* = 2329 (range 14–161) parents. Where parent gender was reported in studies, the majority were female mothers [50–63]. The remaining studies reported on measures provided by a ‘parent’ but did not specify the gender [64–72]. Most children treated across the studies were males (64%). The average age of the child was 5.88 years (SD = 1.82; range 3.81 to 10.35 years). Selection of children into individual studies were based on them having either diagnosis of ADHD, CD or ODD [52, 57, 64, 72, 73], the children having externalising scores in the clinical range [51, 53, 55–59, 66, 68, 70, 71, 73, 74], or elevated symptoms of externalising behaviours that caused parental concern for their child and/or parenting [50, 53, 55, 56, 61, 65–67, 69, 73, 75–77]. Additionally, some studies included additional inclusion criteria: premature birth [59] giftedness [69], at risk for maltreatment [77], or placed in a licenced nonrelative foster home [58].

In relation to the interventions evaluated, five evaluated Parent–Child Interaction Therapy [50, 57–59, 77], eight studies evaluated Incredible Years Parent Program [52, 56, 61, 64, 67, 70, 73, 76], five studies evaluated Triple P Positive Parenting Program [65, 68, 69, 71, 72], three studies evaluated Parent Management Training, the Oregon model [53, 66, 75], one study evaluated ACT Raising Kids Safe Parenting Programme [51], one study evaluated Well Parent Japan [74], and one study evaluated Brief Parent Training [55]. A total of eight studies delivered the intervention in individual-based format [50, 55–57, 59, 74, 75, 77] and 16 studies delivered the intervention in group-based format [51–54, 58, 61, 64–73, 76]. Studies were conducted in a variety of settings including community mental health services/clinics [50, 67, 76], community family services [51, 55, 57, 66, 75], child centres [68, 70], child psychiatric outpatient clinics [52, 61], social service centres and nursey schools [57], child welfare agencies [58], and university clinics [64, 72, 73]. Seven studies did not report the study settings [53, 59, 65, 69, 71, 74, 77]. Most studies compared the intervention group to a waitlist control group (79%). One study compared to an alternative active treatment (Family Creative Therapy) [50], and four compared BPT to service as usual [55, 58, 68, 75].

Only 15 out of 24 studies reported information on the number of treatment sessions attended by parents. Treatment sessions ranged from 1 to 23 sessions. Eight studies reported the mean number of sessions attended [50, 56, 57, 61, 70, 75–77], five reported the percentage (%) of parents attending all or some of the sessions [53, 55, 67, 71, 74], and two reported on the average hours of intervention [55, 66]. Session length ranged from 1 to 5 h.

Instruments used to measure internalising symptoms included the Child Behaviour Checklist (CBCL) [78] and the Strengths and Difficulties Questionnaire (SDQ) [79]. Specifically, 12 studies [50, 53, 57–59, 61, 64, 67, 71, 73, 74, 77] used the CBCL internalising subscale, which combined scores on the anxious/depressed, withdrawn, and somatic complaints scales [78], while two studies [55, 66] used the CBCL anxious/depressed scores. Additionally, two studies [51, 75] used the SDQ internalising subscale calculated from scores on the emotional and peer problems scales [80]. A further eight studies [52, 56, 65, 68–70, 72, 76] used only scores from the SDQ’s emotional problems scale. No studies assessed internalising outcomes using diagnostic instruments. Only two studies [61, 73] reported fathers’ assessment of internalising symptoms separate to the ‘mother’ or ‘parent’ report.

### Study quality: risk of bias

Figures 1, 2 in supplementary information shows risk of bias ratings for the included studies. Studies were judged to include some degree of risk of bias. For example, some studies were unclear on how random sequences were generated to avoid selection bias [61, 73, 77]. Further, information regarding how this sequence was concealed from study personnel was also not specified [53, 56–58, 61, 64, 69, 72–74, 77]. Two studies [53, 69] had evidence of attrition bias as there was no loss to follow up data. All studies appeared to be free from all other sources of possible bias apart from one study [67] which had evidence of recruitment bias from cluster randomisation and one study [73] with conflict of interest.

### Narrative review of child internalising outcomes following BPT

A summary of internalising outcomes following participation in BPT across studies is provided in Table 2. A total of 12 studies reported statistically significant findings that favoured BPT over control for internalising symptoms, with a range of small to large treatment effect as measured by Cohen’s d (*d* = 0.29–1.4), medium to large effects for partial eta or eta squared (*η*2 = 0.084–0.449) and medium for r-squared (*R*^2^ = 0.08). Out of all 24 studies, sixteen used Cohen’s d, three used partial eta-squared or eta-squared, one study used r-squared, and 4 studies did not report an effect size. Interventions that reported statistically significant change in internalising symptoms following treatment were Parent–Child Interaction Therapy [57–59, 77], Brief Parent Training [55], Incredible Years [61, 67, 74, 76], Triple P [71, 72] and Well Parent Japan [74]. One of these studies [55] reported statistically significant findings that favoured BPT over primary care services as usual, while all other studies reported findings that favoured BPT over waitlist control. Ten of these studies evaluated outcomes using the CBCL internalising outcome measure [55, 57–59, 61, 67, 71, 73, 74, 77]. Studies that reported significant findings also had either ‘mother’ or ‘parent’ report on outcomes. Where fathers’ assessment of outcomes was reported, BPT did not significantly reduce internalising symptoms. Three of the four studies coded as having samples of children with high internalising symptoms reported statistically significant findings that favoured intervention over control [57, 61, 72].

The remaining 12 studies did not find statistically significant differences that favoured BPT over control conditions. Eight of these studies evaluated outcomes using the SDQ to measure internalising symptoms [51, 52, 56, 65, 68–70, 75]. Interventions included Incredible Years [52, 56, 64, 70], Triple P [65, 68, 69], Parent Management Training, the Oregon model [66, 75], Parent Management Training [53], and ACT Raising Kids Safe [51]. One study found marginal non-significant differences between Parent–Child Interaction Therapy and Family Creative Therapy [50]. Nine studies that did not find statistically significant differences between BPT and control groups were delivered in group format [51–53, 64–66, 68–70]. No other patterns of findings were identified in relation to study inclusion criteria, child age, or treatment duration.

### Meta-analysis of treatment effects of BPT on internalising outcomes

**Fig. 2:**
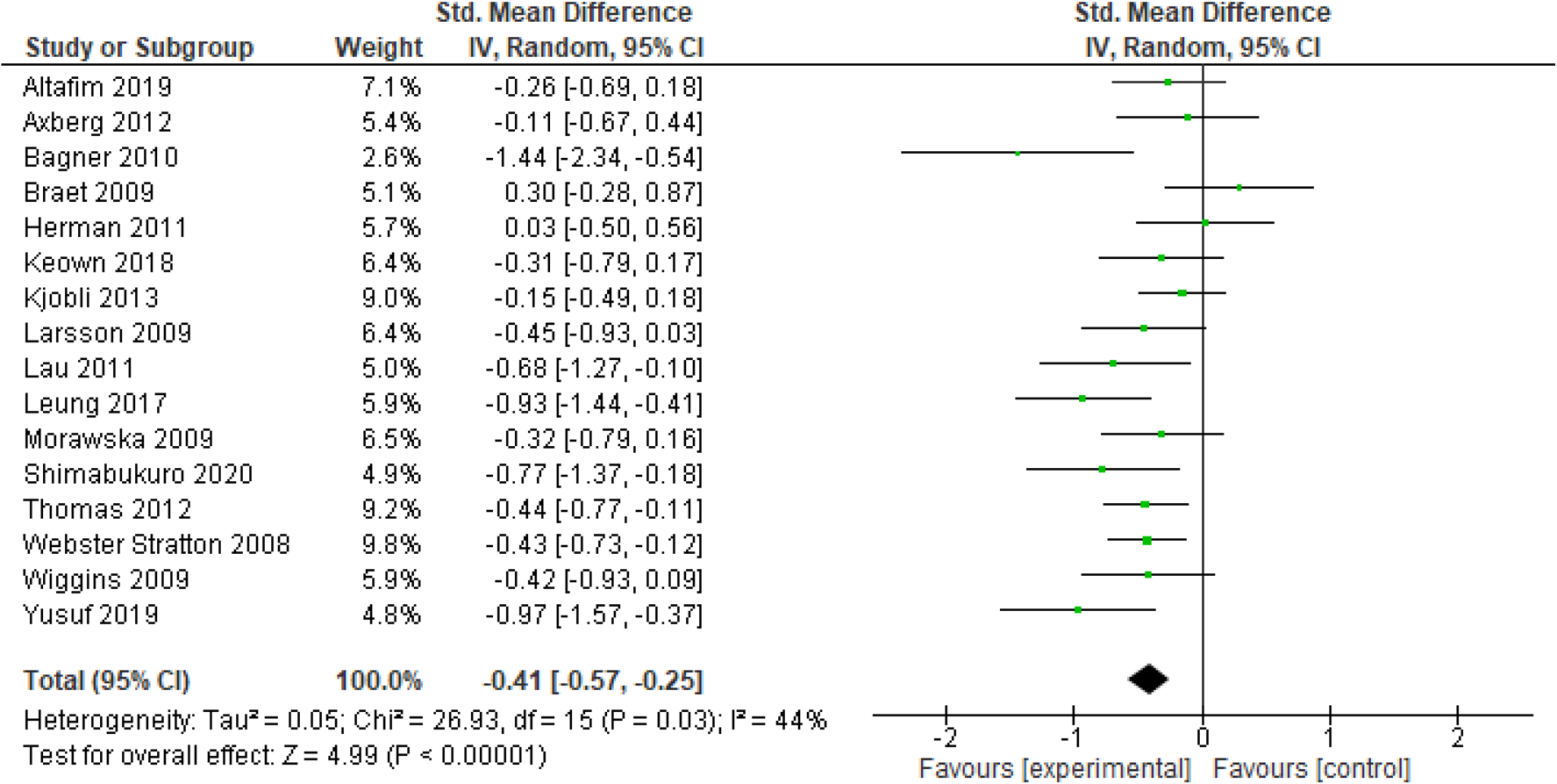
Between-groups meta-analysis and forest plot

A total of 16 studies out of 24 which reported on post-treatment outcomes were included in the meta-analysis ensuring homogeneity in assessing the immediate treatment effect of BPT on internalising symptoms compared to waitlist control. Four studies [56, 68, 70, 76] were removed due to only evaluating follow-up outcomes at 6 and 12 months. A further four studies [50, 55, 58, 75] that compared two active treatments, rather than waitlist controls, were removed. Finally, due to the small number of studies providing fathers’ assessment separately, only the studies that indicated ‘parent’ and ‘mother’ reported scores were included. This also ensured that the children included in the analysis were independent observations. Random-effects meta-analysis (see Fig. 2) demonstrated a statistically significant small post-treatment effect for BPT to reduce internalising symptoms compared to waitlist controls (*g* = − 0.41, 95% CI − 0.57, − 0.25, *z* = 4.99, *p* = 0.00001). Heterogeneity was found to be moderate and significant (*I*^2^ = 44%, *p* = 0.03), with a 95% prediction interval range of − 0.92–0.10. Inspection of the funnel plot and results from Egger’s test of publication bias [49] demonstrated no evidence for publication bias (Fig. 3 in supplementary information).

### Moderation analyses examining the influence of child, treatment, and study factors

Tests for subgroup differences indicated that there were no statistically moderating effects for child age, baseline scores of internalising symptoms, treatment format, measurement scales, programme type, or risk of bias (see Table 3). More specifically, subgroup meta-analyses indicated that BPT had the folowing: (1) statistically significant small treatment effects for studies coded as having younger age group samples; 2–6 years (*k* = 11, *g* = − 0.37, 95% CI − 0.56, − 0.17, *P* = 0.0002, *I*^2^ = 47%) and significant moderate treatment effects for studies coded as having older age group samples; 7–12 years (*k* = 5, *g* = − 0.52, 95% CI − 0.83, − 0.20, *P* = 0.001, *I*^2^ = 49%), (2) small significant effects among studies coded to have low average internalising scores at baseline (*k* = 12, *g* = − 0.34, 95% CI − 0.52, − 0.17, *p* = 0.0001, *I*^2^ = 38%) and moderate significant effects among studies coded to have high average internalising scores at baseline (*k* = 4, *g* = − 0.62, 95% CI − 0.96, − 0.27, *p* = 0.0005, *I*^2^ = 48%), (3) small significant effect among studies with group treatment formats (*k* = 13, *g* = − 0.33, 95% CI − 0.49, − 0.18, *p* = 0.0001, *I*^2^ = 28%), while having a significant large treatment effect among studies with individual treatment formats (*k* = 3, *g* = − 0.82, 95% CI − 1.34, − 0.30, *p* = 0.06, *I*^2^ = 64%), (4) significant small treatment effects among studies that used the CBCL (*k* = 11, *g* = − 0.43, 95% CI − 0.64, − 0.22, *p* = 0.02, *I*^2^ = 54%) and studies that used the SDQ (*k* = 5, *g* = − 0.36, 95% CI − 0.61, − 0.11, *p* = 0.005, *I*^2^ = 20%), (5) a significant small effect was found for studies using the Incredible Years programme (*k* = 5, *g* = − 0.35, 95% CI − 0.56, − 0.14, *p* = 0.001, *I*^2^ = 7%), significant small effect for studies using Triple P (*k* = 4, *g* = − 0.46, 95% CI − 0.74, − 0.19, *p* = 0.001, *I*^2^ = 15%), and a significant large effect for studies using PCIT (*k* = 3, *g* = − 0.82, 95% CI − 1.34, − 0.30, *p* = 0.002, *I*^2^ = 64%), and (6) a significant small effect size was found for studies with no risk of bias (*k* = 6, *g* = − 0.32, 95% CI − 0.57, − 0.08, *p* = 0.002, *I*^2^ = 34%) and for studies with any risk of bias (*k* = 10, *g* = − 0.46, 95% CI − 0.67, − 0.24, *p* = 0.0001, *I*^2^ = 49%). Overall, test for sub-group differences revealed that there were no statistically significant differences between the subgroups.

**Table 3:** Moderation analysis of treatment effect of BPT on internalising outcomes

	*k*	Hedges’ g	95% CI	Heterogeneity (*I*^2^) (%)	Significance	Test for subgroup differences
Internalising scores						
High	4	− 0.62	(− 0.96, − 0.27)	48	*P* = 0.0005	*P* = 0.17
Low	12	− 0.34	(− 0.52, − 0.17)	39	*P* = 0.0001	
Outcome measures						
SDQ	5	− 0.36	(− 0.61, − 0.11)	20	*P* = 0.005	*P* = 0.68
CBCL	11	− 0.43	(− 0.64, − 0.22)	54	*P* = 0.0001	
Treatment format						
Group	13	− 0.33	(− 0.49, − 0.18)	28	*P* = 0.0001	*P* = 0.08
Individual	3	− 0.82	(− 1.34, − 0.30)	64	*P* = 0.0002	
Child age						
2–6	11	− 0.37	(− 0.56, − 0.17)	47	*P* = 0.0002	*P* = 0.43
7–12	5	− 0.52	(− 0.83, − 0.20)	49	*P* = 0.001	
Programme						
IY	5	− 0.35	(− 0.56, − 0.14)	7	*P* = 0.001	*P* = 0.26
TP	4	− 0.46	(− 0.74, − 0.19)	15	*P* = 0.001	
PCIT	3	− 0.82	(− 1.34, − 0.30)	64	*P* = 0.002	
Risk of bias						
No risk of bias	6	− 0.32	(− 0.57, − 0.08)	34	*P* = 0.01	*P* = 0.42
Any risk of bias	10	− 0.46	(− 0.67, − 0.24)	49	*P* = 0.0001	

*k* number of studies, *CI* confidence interval, *p* significance level < 0.05

### Sensitivity analysis

A post-hoc sensitivity analysis was conducted to protect against overestimation of the final effect size due to substantial heterogeneity in treatment effects. Effect sizes and moderation analyses were recalculated by removing the study with the largest SMD in each subgroup in each analysis. Outcomes from calculating the overall treatment effect, tests comparing subgroup effects, and forest plots are provided in Figs. 4–10 in supplementary information. The significance of the overall treatment effect and subgroup effects did not change because of removing the studies with the largest SMD in each subgroup. Further, results of comparing subgroup effects remained the same as the results from moderation analysis reported above.

## Discussion

This review examined the treatment effects of Behavioural Parent Training (BPT) on internalising outcomes among children between 2 and 12 years of age with externalising problems. A total of 12 of 24 studies reported statistically significant findings that favoured BPT over a control or alternative treatment for reducing internalising symptoms. Results from meta-analysis found an overall small significant effect for reducing internalising symptoms following treatment. Sub-group analyses revealed that variations in baseline internalising symptoms, age, measures used, treatment format or programme type did not moderate the overall treatment effect size. However, the current evidence is notably limited by methodological factors. A lack of multi-method and multi-informant measurement of outcomes, alongside heterogeneity across studies and overlooked evaluations of clinical significance, were highlighted as salient limitations of existing research in determining whether BPT is a treatment modality that potentially meets the needs for children with co-occurring externalising and internalising symptoms.

Overall results from the current meta-analysis preliminarily support BPT to significantly reduce internalising symptoms following treatment, with treatment effects within the small range. These results differ from the previous meta-analysis by Buchanan-Pascall et al. [20] which found little evidence to suggest that group-based BPT significantly reduced internalising symptoms following treatment. Differences in treatment modality may partially account for differences, as the current analysis included individual-based BPT which was shown to have descriptively larger effects compared to group-based CBT. Differences in inclusion criteria are also noted, as our analysis required the child to present with clinically elevated externalising symptoms for the study to be included, while Buchanan-Pascall et al. [20] did not impose any criteria on baseline levels of child externalising problems. Taken together, the findings allude to the potential for BPT to reduce internalising problems in clinical samples of children where joint externalising and internalising problems are likely to be more prevalent [9]. It is noted, however, the pooled treatment effect for BPT was descriptively smaller than the effects reported for well-established CBT treatments for internalising problems [40] suggesting the most effective treatment for child internalising problems remains those with components developed for these problems (e.g., exposure).

Results from subgroup analyses indicated that the pooled treatment effect was robust to variations in child, treatment, and study characteristics, as well as study outliers, suggesting improvements in internalising symptoms following BPT are reliable. BPT would therefore be expected to be suitable for most children with co-occurring externalising and internalising problems. Having said that, descriptive differences in the magnitude of effects for specific treatments preliminarily help elucidate the settings for BPT to have optimal utility in reducing internalising problems. For instance, individual-based programmes such as PCIT [26] were indicated to have larger effects compared to other programmes. While the specific reasons for this was not investigated in the current study, we speculate that PCIT delivered in individual format may have specific advantages. For example, PCIT places a strong emphasis on improving the quality of parent–child relationships based on attachment theories of emotion regulation [81]. Additionally, PCIT sessions do not terminate until the parent has sufficiently mastered the skills [82]. As such, BPT programmes that are not exclusively SLT-based, and which include a focus on improving the quality of the parent–child relationship, may provide specific benefits for the amelioration of child internalising symptoms following treatment [83].

While these results appear promising for BPT to have secondary benefits for reducing internalising problems, they are notably tentative given the limited information available from existing trials to accurately determine cross-over effects. One limitation pertains to the assessment of internalising outcomes. For instance, studies included in the review relied on using single-informant methodology, excluding analysis of outcomes reported by different informants including teacher and child self-reports. Most informants were also mothers, which could have led to biased estimates of the treatment effect since our review indicated that positive results were obtained for mothers’ but not fathers’ ratings on treatment outcomes. Additionally, only two main standardised measures were used in individual studies (e.g., CBCL and SDQ), with findings from the narrative review suggesting a trend towards increased likelihood of significant treatment effects where CBCL was used compared to the SDQ. Differences in items may putatively account for some of the inconsistency observed in the results [84]. Included studies also did not consider objective diagnostic assessment by clinicians or determination of whether reductions in internalising symptoms represented a clinically meaningful change following treatment. It therefore remains unclear whether BPT has clinical utility in reducing child internalising problems following treatment. Moderate heterogeneity in effect sizes across studies also suggests that calculations of overall treatment effects remain susceptible to variations in individual study characteristics.

### Future implications for clinical trials evaluating BPT

Significant improvement is required in the scientific rigour and quality of research examining secondary child mental health outcomes from BPT. For instance, there is a paucity of research studies that analyse the effects of BPT on samples of children who have comorbid externalising and internalising problems. Future studies must prioritise comorbid samples of children so that the potential transdiagnostic benefits of BPT programmes can be reliably assessed. Another key limitation of research relates to the generally small samples used in clinical trials. This is exemplified by the contrasting results obtained from our narrative review of individual tests of pre- to post-treatment changes in internalising symptoms across studies versus calculations of pooled treatment effects from meta-analysis. Consistent with previous reviews, findings from the current narrative review found that only half of included studies favoured BPT over a control or alternative treatment for child internalising symptoms despite small significant pooled treatment effects [19]. The contrasting findings by narrative review allude to the problem that previous clinical trials probably did not possess sufficient power to detect small treatment effects. Future clinical trials are recommended to base their sample size estimates to detect secondary treatment effects within the small range.

There is also need for more rigour in assessing secondary outcomes within clinical trials. To surmount limitations of relying on single-informant self-report measures, we recommend going beyond just analysing statistical significance and additionally report diagnostic and clinically meaningful change outcomes to help inform evaluations of the clinical utility of BPT for addressing co-occurring mental health problems. To that end, future meta-analysis of secondary child outcomes will benefit from studies reporting lower-order dimensions of internalising symptoms (i.e., anxiety, depression) to derive an accurate assessment of where gains are made during treatment. Finally, it might be valuable to conduct treatment component analysis to elucidate which components contribute towards the greatest change in child internalising symptoms. For instance, future research may explore whether parenting strategies based on attachment theory (i.e., PCIT) represent key mechanisms of change for internalising outcomes following treatment.

### Implications for clinical practice

We posit that the current results converge with recommendations for practice emerging from the science of personalising psychological interventions [85]. That is, the current findings suggest that BPT has the potential to reduce internalising symptoms for a subset of children referred for the treatment of externalising problems. In line with this, BPT could represent the first line of treatment, with improvements anticipated for both externalising and internalising symptoms. Treatment may then be augmented with disorder-specific interventions if comorbid internalising problems persist, using well-established treatment components developed for internalising problems [86]. This approach to personalising intervention calls for a sequential treatment protocol, whereby children and families receive an evidence-supported intervention for primary externalising problems in the first instance, which is then tailored to address the child’s internalising problems based on treatment response [87]. To that end, developments in transdiagnostic programmes that have a modular design represent a promising direction to enhance the science of personalising interventions for co-occurring child externalising and internalising problems [88]. The MATCH transdiagnostic programme (Modular Approach to Therapy for Children With Anxiety, Depression, Trauma, or Conduct Problems; MATCH-ADTC), for instance, employs a modular design to facilitate personalisation of psychotherapeutic intervention. Modules are drawn from evidence-supported treatments for internalising and externalising disorders, namely cognitive-behavioural therapy and BPT, with accompanying clinical-decision flowcharts to guide treatment [89]. Research studies have shown the potential for superior clinical outcomes from MATCH compared to standard treatment, suggesting sequential treatment protocols may produce incremental benefits compared to standard BPT [90, 91]. We note wider empirical research evaluating sequential treatment protocols involving well-established standard BPT programmes are currently lacking but represent a promising direction for research aimed at optimising treatment outcomes for children with externalising problems [92].

### Strengths, limitations, and future directions from the current review

To our knowledge, this is the first attempt to systematically review and quantitatively analyse the evidence base for the potential benefits of BPT for child internalising symptoms that included individual- and group-based formats. The search strategy followed appropriate PRISMA guidelines [93], while moderation and sensitivity analyses was conducted to ensure analysis was comprehensive and reliable. However, results from this review should be considered in the context of its limitations. In relation to bias, it is possible that the search strategy may have been subject to cultural bias by excluding non-English language studies. It is also possible that eligible studies were overlooked by choosing to report on RCTs rather than grey literature. Moderation analysis was conducted by separating individual studies into 2 or more groups, potentially omitting key findings best identified through continuous measurement. As such, details relating to the child’s age, or the specific number of treatment sessions could not be identified. Future research could look at individual-level data to get a better understanding of these factors. Further, our review was limited to the immediate post treatment effects. Analysis of long-term outcomes would give an insight into how useful BPT is in preventing the continuity or development of internalising problems over time [19]. Finally, the current meta-analysis compared BPT to waitlist control to increase homogeneity in the sample of studies used to calculate treatment effect sizes. It is therefore still unclear whether BPT achieves better outcomes in reducing internalising outcomes compared to other treatments or service as usual. A comparative analysis across treatments is thus warranted.

## Conclusion

Outcomes from this review suggests behavioural parent training (BPT) programmes may have the potential to lead to secondary benefits for reducing internalising symptoms among externalising children [7]. However, notable caution is required as the current evidence remains mixed and the quality of the methods used in the literature also suggests that perhaps not enough evidence exists to make reliable conclusions. Nevertheless, outcomes from the novel use of moderations analysis identified that instead of considering BPT universally reducing internalising symptoms, more careful thought needs to be given to considerations related to baseline levels of internalising symptoms, treatment programme components and format, and the methods used to measure outcomes during treatment, in determining the treatment effects. Further, there was promising evidence that BPT may reduce internalising symptoms among children with elevated symptoms at baseline representing populations with comorbid externalising and internalising symptoms. The future of transdiagnostic benefits is filled with promise for improving efficiency in early interventions for children’s mental health.

### Supplementary Information

Below is the link to the electronic supplementary material.

Supplementary file1 (DOCX 191 KB)
