# Correction to: Does behavioural parent training reduce internalising symptoms (or not) among children with externalising problems? Systematic review and meta-analysis

**DOI:** 10.1007/s00787-023-02201-z

**Published:** 2023-04-03

**Authors:** Christy Bloss, Sophie Brown, Vilas Sawrikar

**Affiliations:** 1 Department of Clinical Psychology, School of Health in Social Science, University of Edinburgh, Edinburgh, UK; 2 Centre of Applied Developmental Psychology, University of Edinburgh, Edinburgh, UK

**Correction to: European Child & Adolescent Psychiatry** 10.1007/s00787-022-02122-3

In the original publication, there were errors in Table 3.

The correct Table 3 is provided below:

**Table 3:** Moderation analysis of treatment effect of BPT on internalising outcomes

	*k*	Hedges’ *g*	95% CI	Heterogeneity (*I*^2^) (%)	Significance	Test for subgroup differences
Internalising scores						
High	4	− 0.62	(− 0.96, − 0.27)	48	*P* = 0.0005	*P* = 0.17
Low	12	− 0.34	(− 0.52, − 0.17)	39	*P* = 0.0001	
Outcome measures						
SDQ	5	− 0.36	(− 0.61, − 0.11)	20	*P* = 0.005	*P* = 0.68
CBCL	11	− 0.43	(− 0.64, − 0.22)	54	*P* = 0.0001	
Treatment format						
Group	13	− 0.33	(− 0.49, − 0.18)	28	*P* = 0.0001	*P* = 0.08
Individual	3	− 0.82	(− 1.34, − 0.30)	64	*P* = 0.002	
Child age						
2–6	11	− 0.37	(− 0.56, − 0.17)	47	*P* = 0.0002	*P* = 0.43
7–12	5	− 0.52	(− 0.83, − 0.20)	49	*P* = 0.001	
Programme						
IY	5	− 0.35	(− 0.56, − 0.14)	7	*P* = 0.001	*P* = 0.26
TP	4	− 0.46	(− 0.74, − 0.19)	15	*P* = 0.001	
PCIT	3	− 0.82	(− 1.34, − 0.30)	64	*P* = 0.002	
Risk of bias						
No risk of bias	6	− 0.32	(− 0.57, − 0.08)	34	*P* = 0.01	*P* = 0.42
Any risk of bias	10	− 0.46	(− 0.67, − 0.24)	49	*P* = 0.0001	

*k* number of studies, *CI* confidence interval, *P* significance level < 0.05

The Original article has been corrected.

