# Supplementary material for: Does behavioural parent training reduce internalising symptoms (or not) among children with externalising problems? Systematic review and meta-analysis
Source: Eur Child Adolesc Psychiatry. 2022 Dec 17;33(8):2485–501. doi: 10.1007/s00787-022-02122-3 (PMC11272747; doi:10.1007/s00787-022-02122-3)
Supplement: Supplementary file 1 — Supplementary file1 (DOCX 191 KB) [file 787_2022_2122_MOESM1_ESM.docx]

**Supplementary information Table 1.** Abstract Screening Tool

| Questions |
| --- |
| Does the title/abstract describe a primary research study as opposed to a review? |
| Does the title/abstract describe the use of a parenting programme as the primary intervention? |
| Does the intervention target behavioural problems/disorders as an outcome? |
| Does the intervention include children aged between 2-12 years old? |

*Notes*. *If one question is a maybe/unsure still an overall yes; *More than one maybe/unsure mark as overall maybe

**Supplementary information Figure 1**. Overall % of Studies’ Risk of Bias Assessment


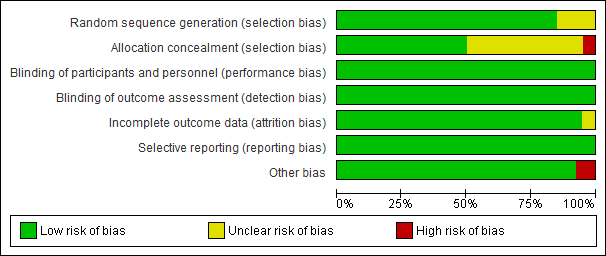


**Supplementary information Figure 2**. Risk of bias assessment


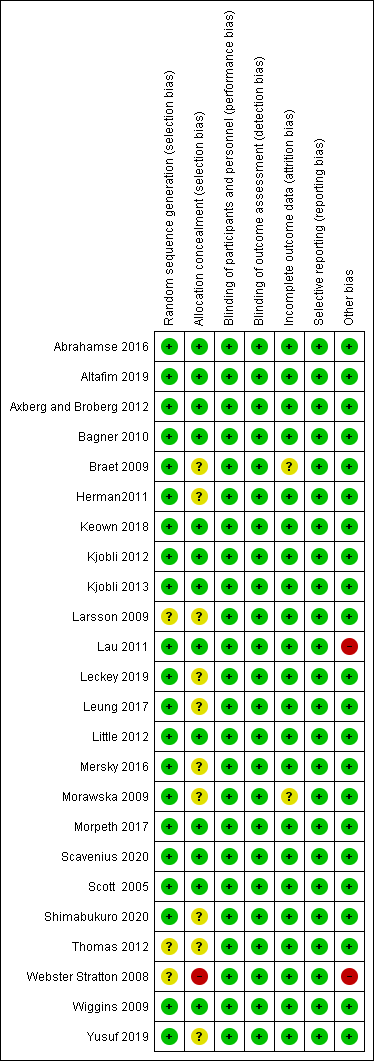


**Supplementary information Figure 3.** Funnel plot of included studies

**
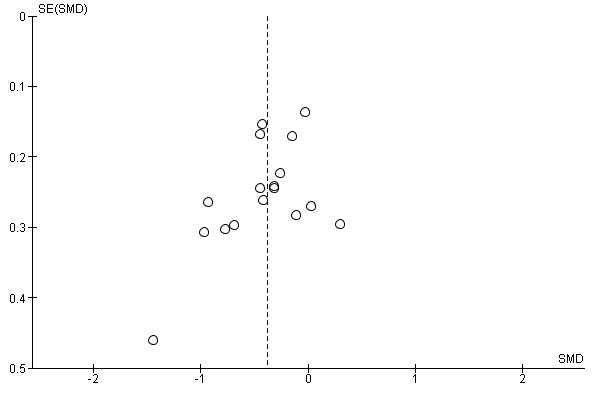
**

Results from Egger’s test of funnel plot asymmetry: β_0_ = .05 (.07), *p*-value = .44, 95% CI (-.09, .20)

**Supplementary information Figure 4**. Sensitivity analysis: Overall treatment effect


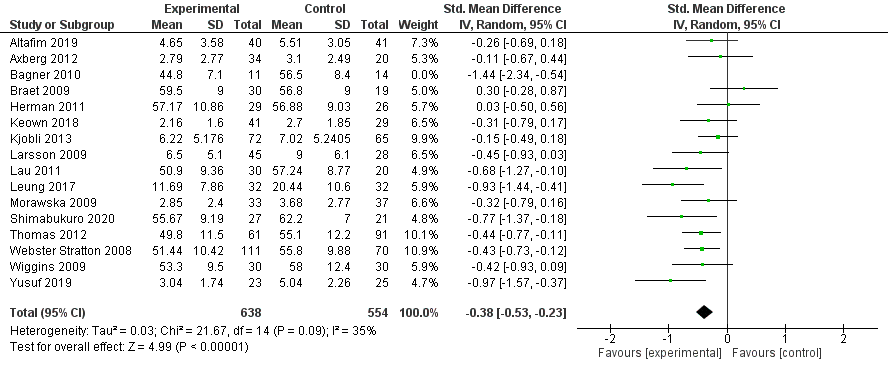


*Notes*. Standard mean difference for Bagner 2010 removed for sensitivity analysis.

**Supplementary information Figure 5**. Sensitivity analysis: Age comparisons


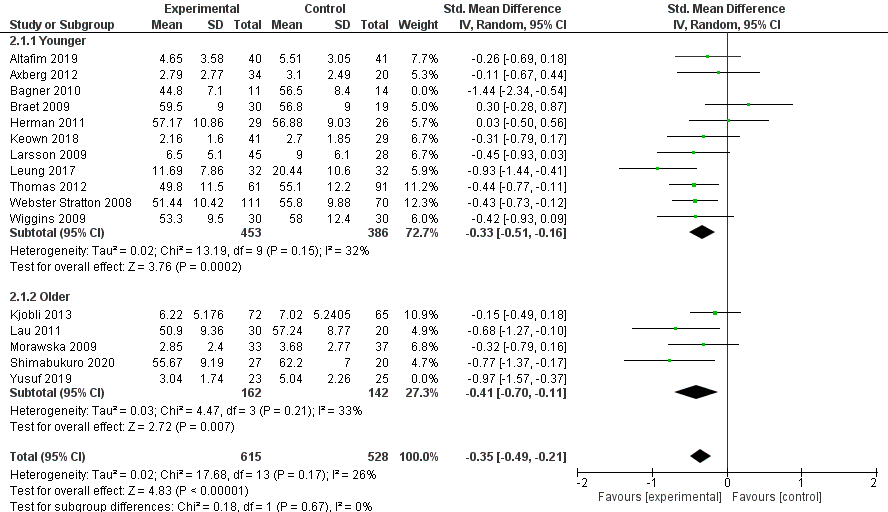


*Notes*. Standard mean difference for Bagner 2010 and Yusuf 2019 removed for sensitivity analysis.

**Supplementary information Figure 6**. Sensitivity analysis: Baseline internalising symptoms


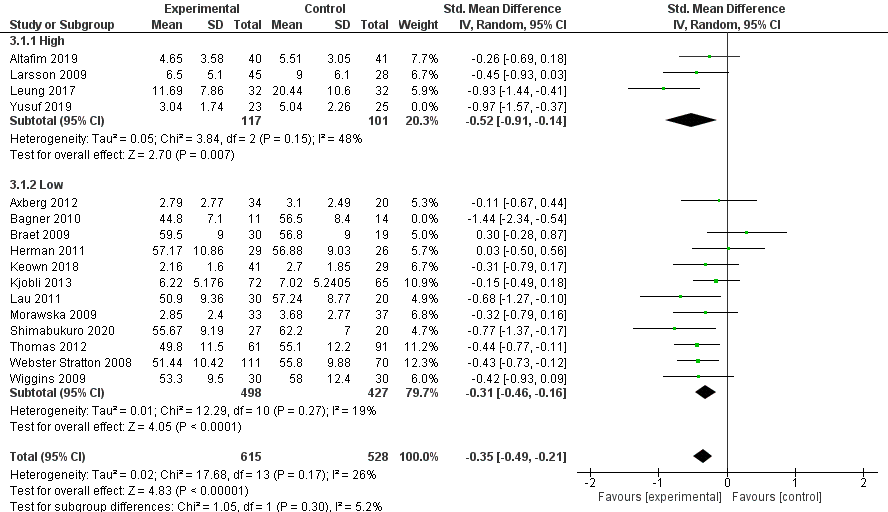


*Notes*. Standard mean difference for Bagner 2010 and Yusuf 2019 removed for sensitivity analysis.

**Supplementary information Figure 7**. Sensitivity analysis: Measures


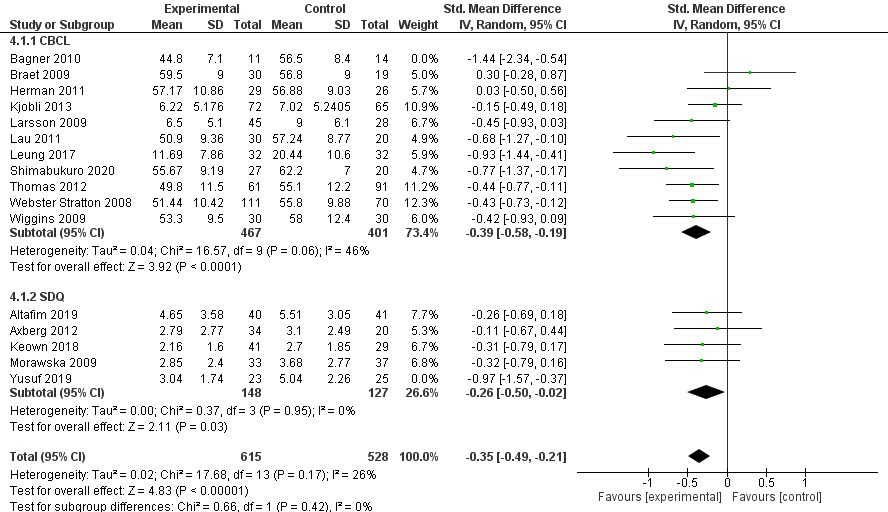


*Notes*. Standard mean difference for Bagner 2010 and Yusuf 2019 removed for sensitivity analysis.

**Supplementary information Figure 8**. Sensitivity analysis: Measures


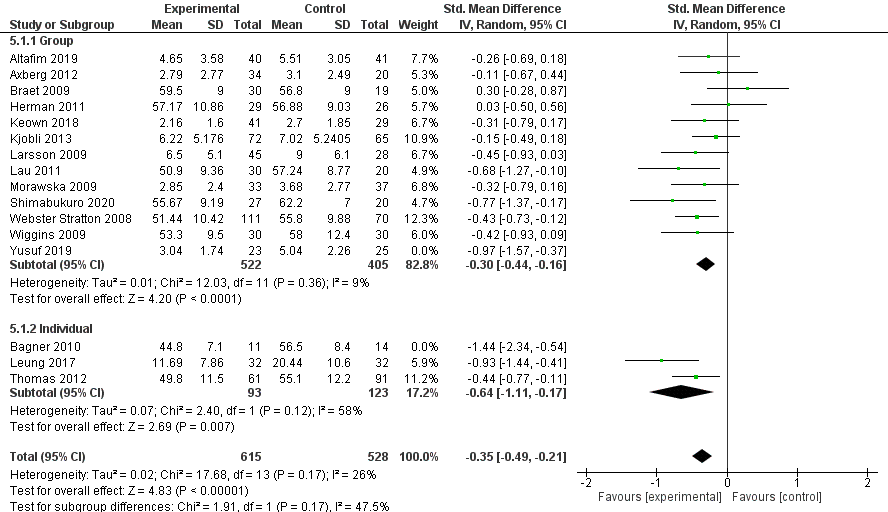


*Notes*. Standard mean difference for Bagner 2010 and Yusuf 2019 removed for sensitivity analysis.

**Supplementary information Figure 9**. Sensitivity analysis: Programme type


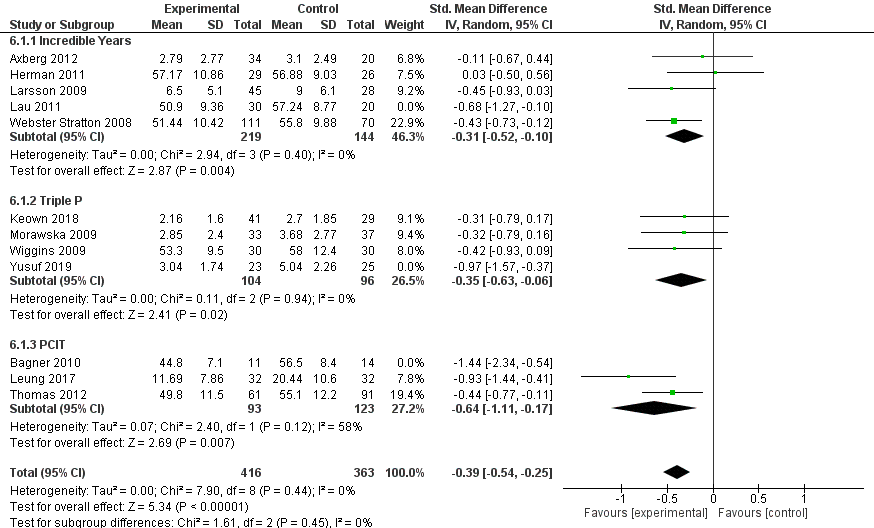


*Notes*. Standard mean difference for Lau 2011, Bagner 2010 and Yusuf 2019 removed for sensitivity analysis.

**Supplementary information Figure 10**. Sensitivity analysis: Risk of bias


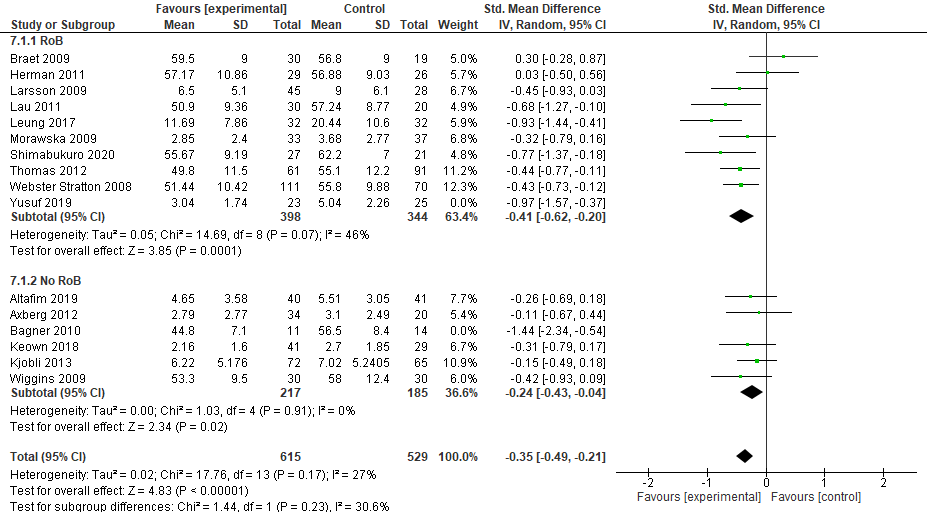


*Notes*. Standard mean difference for Bagner 2010 and Yusuf 2019 removed for sensitivity analysis.
